# Evaluation of unipolar and bipolar nanosecond pulses for calcium electrochemotherapy and immune response

**DOI:** 10.3389/fimmu.2026.1805130

**Published:** 2026-04-20

**Authors:** Eivina Radzevičiūtė-Valčiukė, Augustinas Želvys, Veronika Malyško, Eglė Mickevičiūtė-Zinkuvienė, Paulina Malakauskaitė, Barbora Lekešytė, Jovita Gečaitė, Auksė Zinkevičienė, Vytautas Kašėta, Julita Kulbacka, Joanna Rossowska, Vitalij Novickij

**Affiliations:** 1 Department of Immunology and Bioelectrochemistry, State Research Institute Centre for Innovative Medicine, Vilnius, Lithuania; 2 Faculty of Electronics, Vilnius Gediminas Technical University, Vilnius, Lithuania; 3 Department of Molecular and Cellular Biology, Faculty of Pharmacy, Wroclaw Medical University, Wroclaw, Poland; 4 Hirszfeld Institute of Immunology and Experimental Therapy, Polish Academy of Sciences, Wroclaw, Poland

**Keywords:** bipolar, calcium electroporation, cancer, electrochemotherapy, immunomodulation, *in vivo*, nanosecond, unipolar

## Abstract

Calcium electrochemotherapy (CaECT) is an effective alternative to standard chemotherapeutic treatments, utilizing intracellular delivery of supraphysiological calcium concentrations to induce cell death. High-frequency sub-microsecond bursts can be successfully used for CaECT, however, bipolar waveforms have not yet been characterized in this context, potentially offering better impedance mitigation and more uniform treatment when compared to unipolar procedures. Therefore, this study evaluated the feasibility of unipolar and symmetric bipolar sub-microsecond pulses (7 kV/cm × 300 ns × 250, 1 MHz) for CaECT including their capacity to modulate antitumor immunity in a moderately immunogenic murine breast cancer model. Standard microsecond protocol (1.5 kV/cm × 100 μs × 8 pulses at 1 Hz) was used as a reference. *In vitro* data revealed that a bipolar cancellation phenomenon when symmetric bipolar pulses were applied, whereas this effect was not detected *in vivo*. CaECT treatment induced systemic immune alterations across electroporation groups, including increased CD4^+^ and CD8^+^ memory T-cell populations in the spleen and reduced CD4^+^ regulatory T cells and myeloid-derived suppressor cells in tumor-draining lymph nodes. Unipolar nanosecond pulses showed a clearer increase in central memory T-cell populations, while bipolar pulses were associated with pronounced modulation of lymph-node immune composition. It is shown that bipolar cancellation phenomenon is not necessarily triggered *in vivo*, which was predicted by *in vitro* data.

## 1 Introduction

Electrochemotherapy (ECT) is a rapidly emerging cancer treatment method that combines electroporation (EP) with standard chemotherapeutic drugs, such as bleomycin and cisplatin. EP is triggered by external pulsed electric fields (PEFs), which induce cell membrane permeabilization and enable the delivery of initially cell membrane non-permeable ions or molecules into the cell (1). While bleomycin and cisplatin dominate the field, research on EP with other drugs (doxorubicin, vinorelbine, etc.) or ions (e.g., calcium) is constantly being performed to ensure better treatment outcomes (2–4).

Calcium electroporation, or calcium electrochemotherapy (CaECT), is an innovative cancer treatment based on the cytotoxic effects of intracellular elevated calcium ion concentrations, facilitating the delivery of calcium via electroporation-induced membrane pores (5–7). Increased cellular calcium uptake induces tumor cell death owing to the pivotal role of calcium ions in many vital cellular processes, including the regulation of transcription, metabolism, proliferation, muscle contraction (8–10), ATP and mitochondrial homeostasis (7, 11–14). Furthermore, cell death due to CaECT induces immunogenic cell death (ICD), leading to the release of danger-associated molecular patterns (DAMP) (15, 16) such as ATP, High Mobility Group Box 1 protein (HMGB1), and mitochondrial ROS etc. (7, 14, 17–19) The release of these molecules stimulates a local and systemic immune response, aiding in the regression of distant metastases.

The effectiveness of CaECT in cancer treatment has been proven in *in vitro* and *in vivo* cancer models (5, 7, 12, 20–22), veterinary studies (23, 24) and clinical trials (25–29). A murine colon cancer model study on calcium electrochemotherapy reported induction of immunological memory and pro-inflammatory cytokine secretion (22). Our previous study reported that combining irreversible electroporation with calcium boosted splenic T cell numbers and reduced suppressor cell subsets (30). Guo et al. reported that nanosecond pulsed electric fields (nsPEFs) in the 4T1 murine model induce an immune memory response by increasing CD4^+^ effector memory and CD8^+^ central memory lymphocytes, which exhibit high cytotoxicity due to IFNγ production (31). Thus, combining CaECT with other immunotherapies could boost the treatment effectiveness and open new possibilities for cancer therapy. Lisec et al. demonstrated the combination of bleomycin or calcium ECT with gene electrotransfer (GET) of a plasmid encoding interleukin-12 (IL-12) in two different murine cancer models: poorly immunogenic murine melanoma B16-F10 and moderately immunogenic murine mammary carcinoma 4T1. In this study, a higher count of CD8^+^ tumor-infiltrating lymphocytes (TILs) and a significantly increased number of natural killer (NK) cells were reported after combinatorial therapies with IL-12 GET with either calcium or standard chemotherapeutics ECT in both tumor models, with overall reported enhanced antitumor immunity (32). Clinical studies of melanoma have shown that CaECT induces a systemic immune response and abscopal effect, achieving complete remission in both treated and untreated metastases (28, 29).

In EP-based clinical treatments, ECT protocols follow the European Standard Operating Procedures of Electrochemotherapy (ESOPE), using 8 × 100 μs pulses with amplitudes between 1 kV/cm and 1.5 kV/cm (33). Although this cancer treatment modality is reported to be safe and effective, undesirable side effects have been reported, including Joule heating (34, 35), pain and muscle contractions (36), products of electrolysis (37) and inhomogeneous treatment (38). Many of these challenges can be solved with the shorter pulses (nanosecond; nsPEFs) and higher-frequency electroporation (39–42). High-frequency bursts improve gene and drug electrotransfer by maintaining residual transmembrane potential throughout the burst (4, 43, 44). Recent studies demonstrated that bleomycin, doxorubicin and cisplatin-based nsECTs can be as effective and in some cases even better than the standard clinically applicable microsecond procedures (μsECT) (4, 37, 45, 46). However, sub-microsecond bipolar pulses are currently limited to irreversible electroporation, with minimal research on their ECT applicability (19, 47).

Bipolar sub-microsecond pulses are of particular translational interest because their alternating polarity reduces neuromuscular stimulation and electrode corrosion, making them suitable for sensitive anatomical locations. Additionally, higher frequency component (when compared to unipolar pulses) also potentially contributes to impedance mitigation and a more uniform electric field distribution (48, 49). Although *in vitro* assays report reduced dye uptake with symmetric bipolar waveforms (associated with bipolar cancellation (BPC) (50)), *in vivo* outcomes depend on tissue conductivity (51, 52), vascular responses (53), immune engagement (54) – factors not captured by dye permeability measurements. The effects of BPC were mostly observed in various *in vitro* cell cultures (55–58), with limited data on *in vivo* bipolar ECT. Moreover, calcium-induced regulated cell death and immunogenic signaling can be triggered at relatively low permeabilization thresholds (59), suggesting that bipolar sub-microsecond CaECT may remain therapeutically potent despite modest *in-vitro* permeability.

Currently, there are no *in vivo* studies that have explored and compared unipolar and bipolar high-frequency sub-microsecond CaECT. To address this gap, in this study we have evaluated the feasibility of unipolar and bipolar 300-ns bursts (7 kV/cm × 300 ns × 250 pulses at 1 MHz) for CaECT including the comparison of the protocols for immune response modulation. A standard μsECT protocol (1.5 kV/cm × 100 μs × 8 pulses at 1 Hz) was used as a reference to ensure consolidation of knowledge and comparison with established procedures.

## 2 Materials and methods

### 2.1 Pulsed power setup and protocols

A square-wave unipolar and bipolar pulse generator developed at Vilnius Gediminas Technical University (Vilnius, Lithuania) was used (60, 61). Bipolar generator is capable of forming high frequency pulses in a broad range of parameters (65 ns–100 ns, with pulse repetition frequency up to 5 MHz), while unipolar pulse generator creates square wave pulses of 100 ns to 1 ms durations with 1 Hz to 3.5 MHz repetition frequency, supporting voltages up to 3 kV and currents up to 40 A.

**Figure 1:**
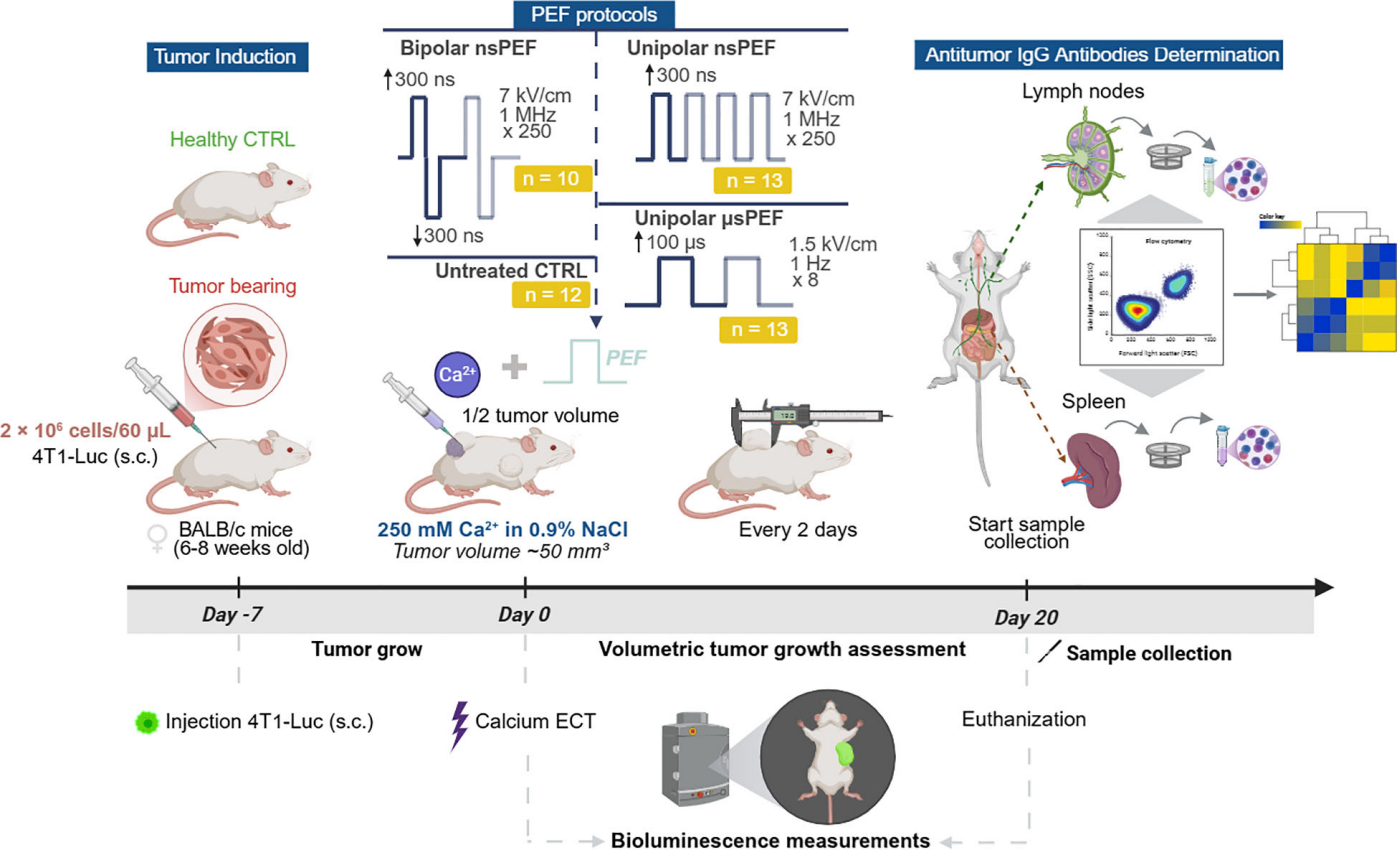
Schematic experimental design.

In this study, we employed two nanosecond pulsed electric field protocols: unipolar (nsCaECT) and symmetrical bipolar (nsCaECT symmetric). The nsCaECT protocol consisted of positive 7 kV/cm amplitude pulses with a 300 ns duration (↑300 ns). In contrast, the nsCaECT symmetric protocol included both positive and negative 7 kV/cm amplitude pulses, each with a 300 ns duration (↑300 ns + ↓300 ns). Both protocols involved the delivery of 250 pulses at a repetition frequency of 1 MHz. As a reference, the µsECT protocol was used, which followed the ESOPE protocol (1.5 kV/cm × 100 µs × 8, 1 Hz) to ensure results comparability with established knowledge in the field. A summary of electroporation protocols is shown in **Figure 1**. Three different PEF protocols were used in this study.

### 2.2 Cell lines

4T1 (ATCC-CRL-2539) is a murine mammary carcinoma cell line derived from BALB/c mice. Previously, we described the development of a luminescent 4T1 cell line using nanosecond-duration electrotransfection methodology. The established luciferase-expressing cell line was named 4T1-Luc (62). Cells were cultured in RPMI 1640 medium supplemented with glutamine, 10% of fetal bovine serum (FBS), and antibiotics (100 U/mL of penicillin, 100 mg/mL of streptomycin) at 37 °C in a humidified atmosphere containing 5% CO_2_. Cell culture reagents were obtained from Gibco (Thermo Fisher Scientific, Waltham, MA, USA). The cell lines were tested for mycoplasma contamination using the MycoBlue^®^ Mycoplasma Detection Kit (Vazyme, Nanjing, China).

### 2.3 Permeability

Cells were detached from the cultivation dish using Trypsin-EDTA solution (Thermo Fisher Scientific, Waltham, MA, USA), collected by centrifugation, and resuspended in electroporation buffer (HEPES 10 mM, sucrose 250 mM, MgCl_2–_1 mM, pH 7.2) at a concentration of 2 × 10 (6) cells/mL. The cells in the electroporation buffer were mixed with Yo-Pro1 (YP, Sigma-Aldrich, St. Louis, MO, USA) to achieve a final concentration of 1 μM. Then, 60 μL of the solution was transferred into an electroporation cuvette and treated using different EP protocols. After 3 min of incubation at room temperature, the cells were transferred into a 96-well round-bottom plate (Nunc, Sigma-Aldrich, St. Louis, MO, USA) and 150 μL of 0.9% NaCl solution was added. Immediately thereafter, the cells were analyzed using a BD Accuri C6 flow cytometer (BD Biosciences, San Jose, CA, USA). YP1 fluorescence was detected using the Channel FL1 (533/30 nm BPF). The results were analyzed using the FlowJo software (BD, Becton Drive Franklin Lakes, NJ, USA).

### 2.4 Viability assessment

A 4T1-luc cell suspension in electroporation buffer was prepared at a concentration of 1.5 × 10 (6) cells/mL, with or without 5 mM CaCl_2_. Electroporation-affected cells (25 μL) were transferred to a 96-well flat-bottom plate. Following 10 minutes incubation, cell growth media with supplements was added to a final volume of 200 μL in each well. Untreated cells were used as controls for data normalization. At the end of the experiment, the plates were transferred to an incubator at 37 °C and 5% CO₂ for 24 h. The following day, the cell viability was measured using metabolic PrestoBlue assay (Thermo Fisher Scientific, Waltham, MA, USA). Initially, all the wells were gently washed twice with phosphate-buffered saline (PBS), and then 150 μL of PBS and 5 μL of cell viability reagent were pipetted into each well, followed by a 2-hour incubation. Fluorescence (Ex. 540/20 nm; Em. 620/40 nm) were measured using a Synergy 2 microplate reader and Gen5 software (BioTek, Shoreline, WA, USA).

### 2.5 Tumor induction and electroporation

For *in vivo* tumor induction, cells were cultured in the exponential phase and harvested in RPMI 1640 medium without supplements, at a density of 2 × 10 (6) cells/60 μL per mouse. 4T1-Luc cells were injected subcutaneously (s.c.) into the left flank to establish tumors. When tumors reached volumes of approximately 50 mm³ (day 0), mice were randomly assigned to experimental groups and treatment was applied. Furthermore, this study included healthy mice that were tumor-free and age-matched, serving as the healthy mice control group. Other potential confounders were not specifically controlled. The number of animals in each experimental group was as follows: bipolar nsPEF (n = 10), unipolar nsPEF (n = 13), unipolar µsPEF (n = 13), and untreated control (n = 12).

Before electroporation mice’s backs were shaved, depilated with 8% Na_2_S solution, and rinsed with water. In the calcium electrochemotherapy (CaECT) group, mice received a single intertumoral injection of a 250 mM calcium chloride solution in 0.9% NaCl prior to treatment (approximately half of the tumor volume). EP was delivered immediately after i.t. injection of the calcium solution. The pulses were delivered using adjustable parallel plate stainless-steel electrodes, compressing the tumor between the flat electrodes (with a 3–4 mm gap) lubricated with EEG and ECG Transound gel (EF Medica Srl, Italy) to ensure good contact between the skin and electrodes. The distance between electrodes was adjusted according to the tumor size to ensure stable positioning and adequate electrical contact. The electric field distribution produced by similar electrode configurations has been previously investigated by our group using numerical simulations (38, 63). Depending on the gap between the electrodes the generator charging voltage was adjusted accordingly to ensure voltage/gap ratio to match the electric field amplitudes of three applied protocols described previously. The waveform was measured/controlled using a TAO3000 oscilloscope (Owon Technology, Ontario, Canada) and a 1:100 P4250 probe (Cleqee, Shenzhen, China). Throughout the treatment, mice were maintained under inhalation anesthesia using 1.5 – 2.0% isoflurane administered via an anesthesia system connected to an oxygen supply. The general scheme of the study design and the EP protocol used are presented in **Figure 1**.

All *in vivo* experimental procedures with laboratory animals were conducted following State Food and Veterinary Service consent (Approval No. G2-226) and strictly in accordance with the Guide for the Care and Use of Laboratory Animals. Female BALB/c mice, aged 6–8 weeks, were bred and housed in the mouse facility of the State Research Institute Centre for Innovative Medicine (Vilnius, Lithuania). Throughout the study, the health of the mice was closely monitored, and measurements were taken three times per week. Tumor size dynamics were measured using a digital caliper and calculated based on a methodology established in previous research (64).

### 2.6 *In vivo* bioluminescence assay

The luminescence of the established tumors was captured using the IVIS Spectrum and Living Image Software (Caliper/Perkin Elmer, Akron, OH, USA). Animals were injected intraperitoneally with 150 µL of D-luciferin (15 mg/mL; Promega, Madison, WI, USA) in PBS 10–15 min prior to imaging. The amount of bioluminescence correlates directly with the number of live and actively proliferating 4T1-Luc cells. Images were captured while the animals were under anesthesia using a mixture of 2% isoflurane and oxygen gas. Luminescence was expressed as photons/s/region of interest (ROI) minus background luminescence for a similarly sized region.

### 2.7 Flow cytometry

Immune system organ (spleen and lymph node) single-cell suspensions were prepared by mashing them through a cell strainer (70 µm) and further centrifuged at 300 × g for 5 min at room temperature (RT). Splenocytes were treated with 0.16 M NH_4_Cl ammonium chloride for 10 min to lyse the mouse erythrocytes. All prepared single-cell suspensions were washed with phosphate-buffered saline (PBS) and then incubated with anti-FcR (Fc-Block) for at least 10 min on ice to block non-specific binding of antibodies. Cell surface staining was performed by incubating 1 × 10 (6) of cells in 20 μL of flow cytometry buffer (FACS buffer; 2% FBS and 0.1% NaN_3_ in PBS) with a 20 μL mixture of desired antibodies, followed by 30 min incubation on ice, away from light. The cell populations were distinguished using various sets of fluorochrome-labeled antibodies and fluorescent dyes. The antibodies used, gating, and analysis strategies are presented in the **Supplementary Data** (**Supplementary Table 1**; **Supplementary Figures 1**–**3**). Stained cells were analyzed using a BD FACS Aria III instrument (BD Biosciences, San Jose, CA, USA). The acquired data were analyzed using the FlowJo software.

### 2.8 Antitumor IgG antibodies determination

Antitumor IgG antibodies against both intracellular and extracellular 4T1 cell antigens in murine blood sera were assessed based on a previously described protocol (54). Briefly, the cells were fixed with 2% paraformaldehyde (PFA) in PBS buffer for 10 min at 37 °C. After incubation, the cells were washed and centrifuged at 300 × g for 5 min at 4 °C, then permeabilized with ice-cold 0.2% Triton X-100 in PBS for 9 min on ice. Immediately after, the cells were resuspended in FACS buffer, centrifuged at 300× g for 5 min at 4 °C, diluted in Fc-Block at density 0.3 × 10 (6) cells per sample, and filtered using a 70 µm cell strainer. Next, the cells were incubated with diluted mouse serum for 1 h, followed by washing with PBS, and centrifuged at 300 × g for 5 min at a temperature of 4 °C. Afterwards, the samples were incubated with anti-mouse IgG AF488 antibodies, followed by 30 min of incubation on ice, away from light. As a negative control, the cells were incubated with goat anti-mouse IgG AF488 antibodies, without the addition of mouse serum. Measurements were performed using an Amnis FlowSight cytometer (Amnis Luminex/Millipore Sigma, Burlington, MA, USA). The acquired data were analyzed using ImageStream IDEAS software (Amnis Luminex/MilliporeSigma, Burlington, MA, USA).

### 2.9 Statistical analysis

Prior to statistical analysis, data distribution was assessed for normality using the Shapiro–Wilk test. For *in vitro* data analysis, one-way analysis of variance (ANOVA) was performed (p < 0.05), and when ANOVA indicated statistically significant differences, Tukey’s HSD multiple comparison test was applied. *In vitro* experiments were conducted at least three times independently, and treatment efficiency is reported as mean ± standard deviation.

Principal component analysis (PCA) was performed separately for lymph node and spleen datasets to evaluate multivariate immune-profile changes across treatment groups. Prior to analysis, samples with missing values were removed or linearly imputed, and all markers were z-score standardized. PCA was conducted using the covariance matrix, and PC1–PC2 score plots with 95% confidence ellipses and loading vectors were generated to identify markers contributing to group separation. Complementary hierarchical clustering heat maps (z-score standardized, Euclidean distance, Ward’s linkage) were used to visualize coordinated immune marker changes across groups.

For non-normally distributed data, a non-parametric Kruskal–Wallis test was performed, followed by Dunn’s *post hoc* multiple comparison test with correction to determine significant differences in spleen weight and immune cell subpopulations among treated and untreated groups. Survival differences between groups were assessed using Kaplan–Meier analysis with the log-rank test.

A p value < 0.05 was considered statistically significant (ns p ≥ 0.05; * p < 0.05; ** p < 0.005; *** p < 0.0005). All statistical analyses and graphical visualization were performed using GraphPad Prism 8 (GraphPad Software, San Jose, CA, USA).

## 3 Results

### 3.1 *In vitro* unipolar microsecond and nanosecond CaECT reduce cell viability

**Figure 2:**
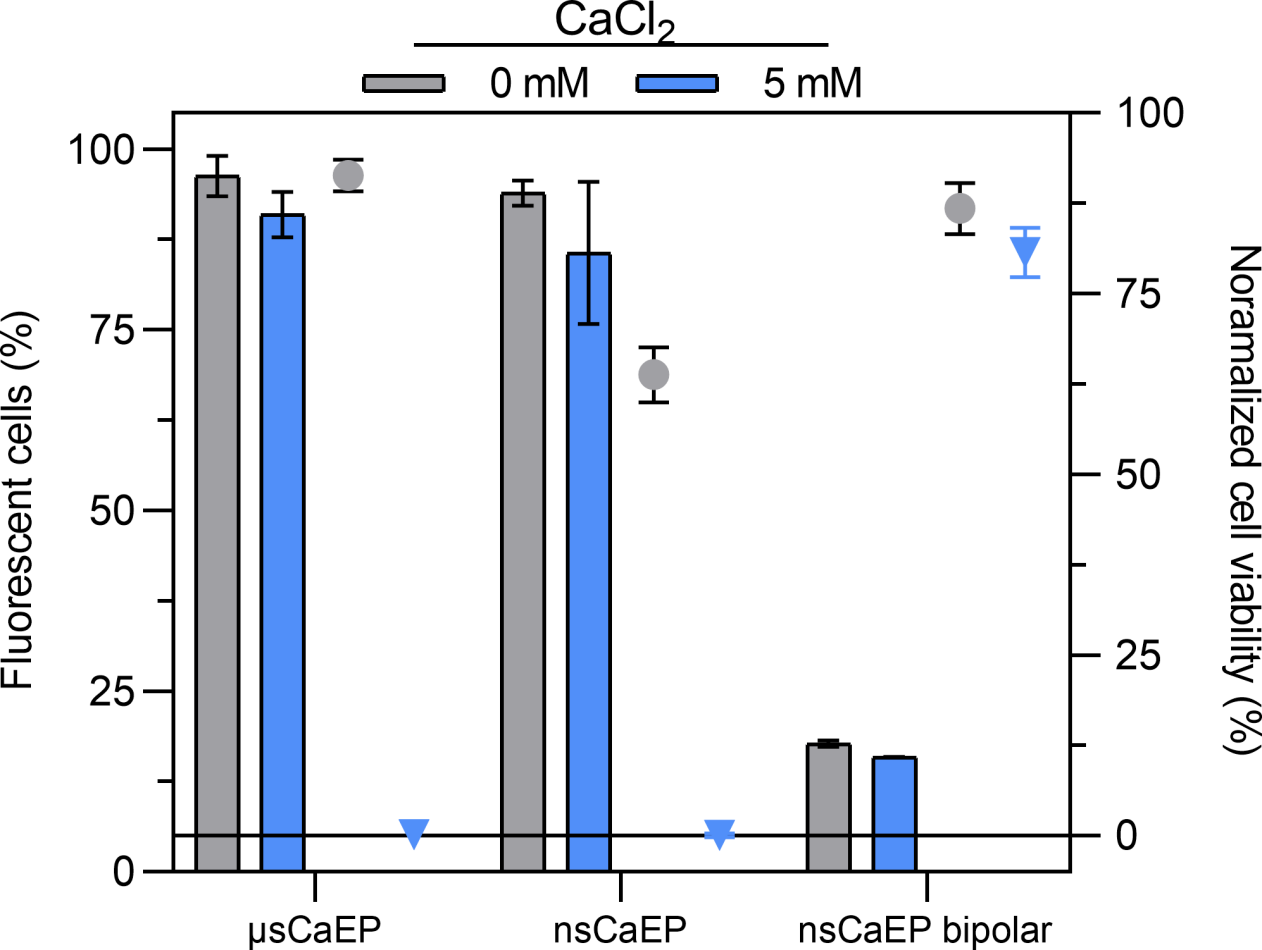
Detection of cell membrane permeabilization (columns) and cell viability (dots and triangles) without (gray) and with 5 mM calcium (blue), where μsCaECT – 1.5 kV/cm × 100 µs × 8, 1 Hz; nsCaECT – 7 kV/cm × ↑300 ns × 250, 1 MHz; nsCaECT symmetric – 7 kV/cm × ↑300 ns + ↓300 ns × 250, 1 MHz. Data are presented as mean ± standard deviation (SD), and error bars represent standard deviation.

First, the effects of the high-frequency nanosecond bipolar and unipolar ECT protocols involved in the study were evaluated *in vitro* in the context of cell membrane permeabilization and viability post-electroporation, both with and without additional calcium. Cell membrane permeabilization was assessed based on the uptake of a fluorescent marker (YP), and the viability of 4T1 cells after treatment was determined using a metabolic activity assay. The results are shown in **Figure 2**.

As shown in **Figure 2** (columns), the number of fluorescent cells slightly decreased with calcium (5 mM), although not significantly. Unipolar micro- and nanosecond protocols trigger high cell membrane permeabilization (> 80%), whereas bipolar nanosecond symmetric pulses induce less than 25% permeabilization, due to the BCP phenomenon regardless of calcium.

Additionally, the viability of 4T1 cells 24 h after treatment was evaluated (**Figure 2**; dots and triangles). The results showed that calcium significantly reduced cell viability (no viable cells) with unipolar micro- and nanosecond indicating successful CaECT. In case of bipolar symmetric pulses, viability exceeded 75%, regardless of calcium due to BPC phenomenon. Nevertheless, we have tested all three parametric protocols *in vivo* to understand if the reported effects are transferable.

### 3.2 Calcium ECT delays tumor growth

**Figure 3:**
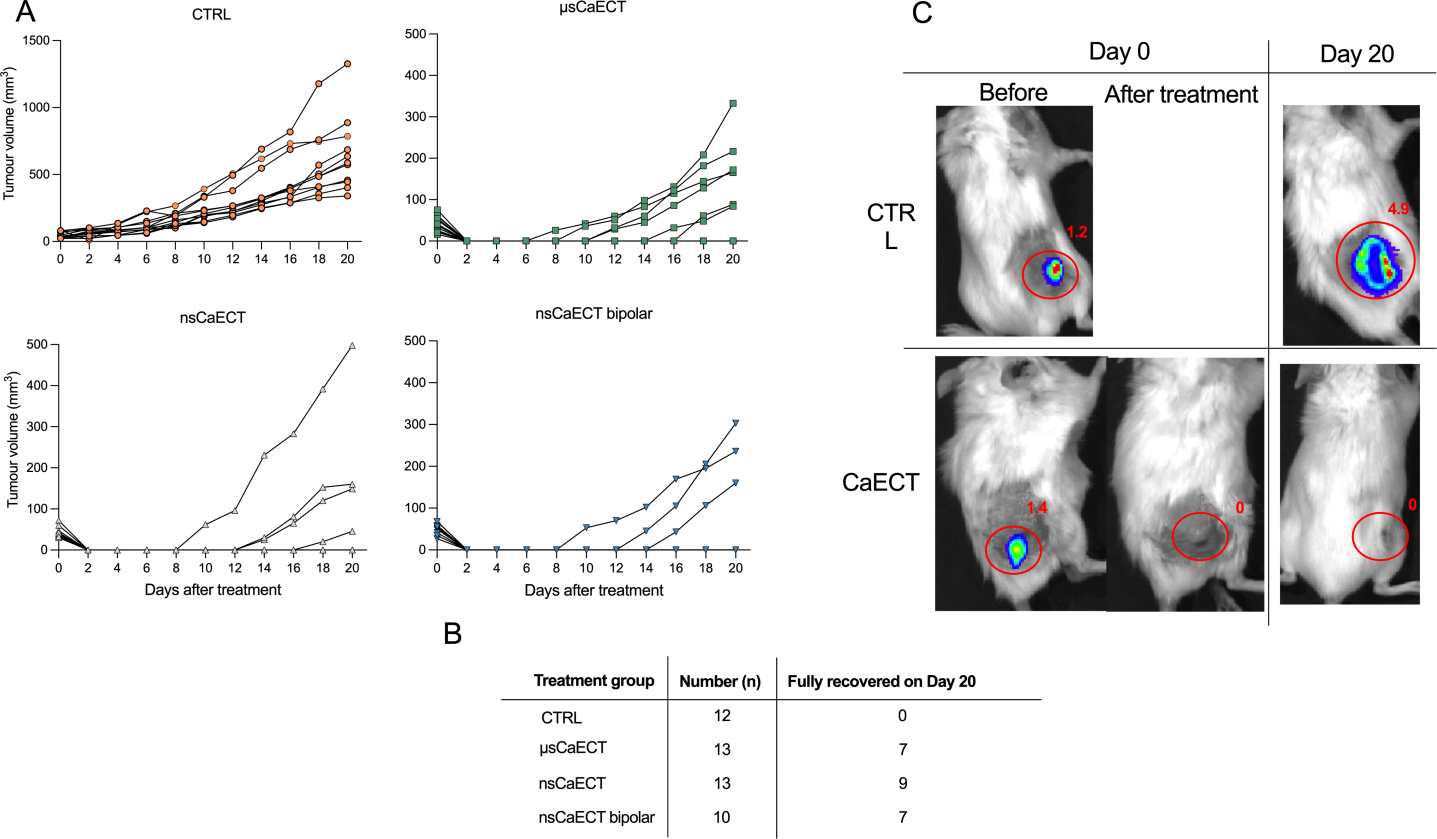
**(A)** Volumetric tumor growth assessment of individual animals in each group; **(B)** Table showing the number of mice in each group and the number of fully recovered mice 20 days after treatment; **(C)** Representative BALB/c murine tumors luminescence images received from an IVIS Spectrum device and Living Image software, where total amount of (p/s × 10 (6)) are marked in red; CTRL – untreated tumor-bearing mice; μsCaECT – 1.5 kV/cm × 100 µs × 8, 1 Hz; nsCaECT – 7 kV/cm × ↑300 ns × 250, 1 MHz; nsCaECT symmetric – 7 kV/cm × ↑300 ns + ↓300 ns × 250, 1 MHz.

Untreated tumor-bearing control (CTRL) and healthy mice groups were included in the study as control groups. First, we evaluated the effect of CaECT treatment on tumor growth in BALB/c mice (**Figure 3**). As shown in **Figure 3A**, tumor growth was reduced in all CaECT treatment groups when compared with untreated tumor-bearing mice, indicating that CaECT slows tumor growth, regardless of whether unipolar or bipolar pulses were applied, which was not the case *in vitro*. Individual animal responses varied, likely due to the non-uniform electric field distribution affected by tumor morphology, tissue heterogeneity, and non-invasive plate electrodes.

At the end of the experiment (day 20), majority of mice in the CaECT treatment groups fully recovered, with no tumors. Standard microsecond CaECT (μsCaECT) resulted in over 50% recovery, whereas sub-microsecond CaECT with unipolar and bipolar pulses induced approximately 70% full recovery. The full recovery of animals incl. the lack of metastases was confirmed by bioluminescence (representative images are shown in **Figure 3C**).

### 3.3 Changes in immune cells of the splenic and tumor-draining lymph nodes following CaECT

**Figure 4:**
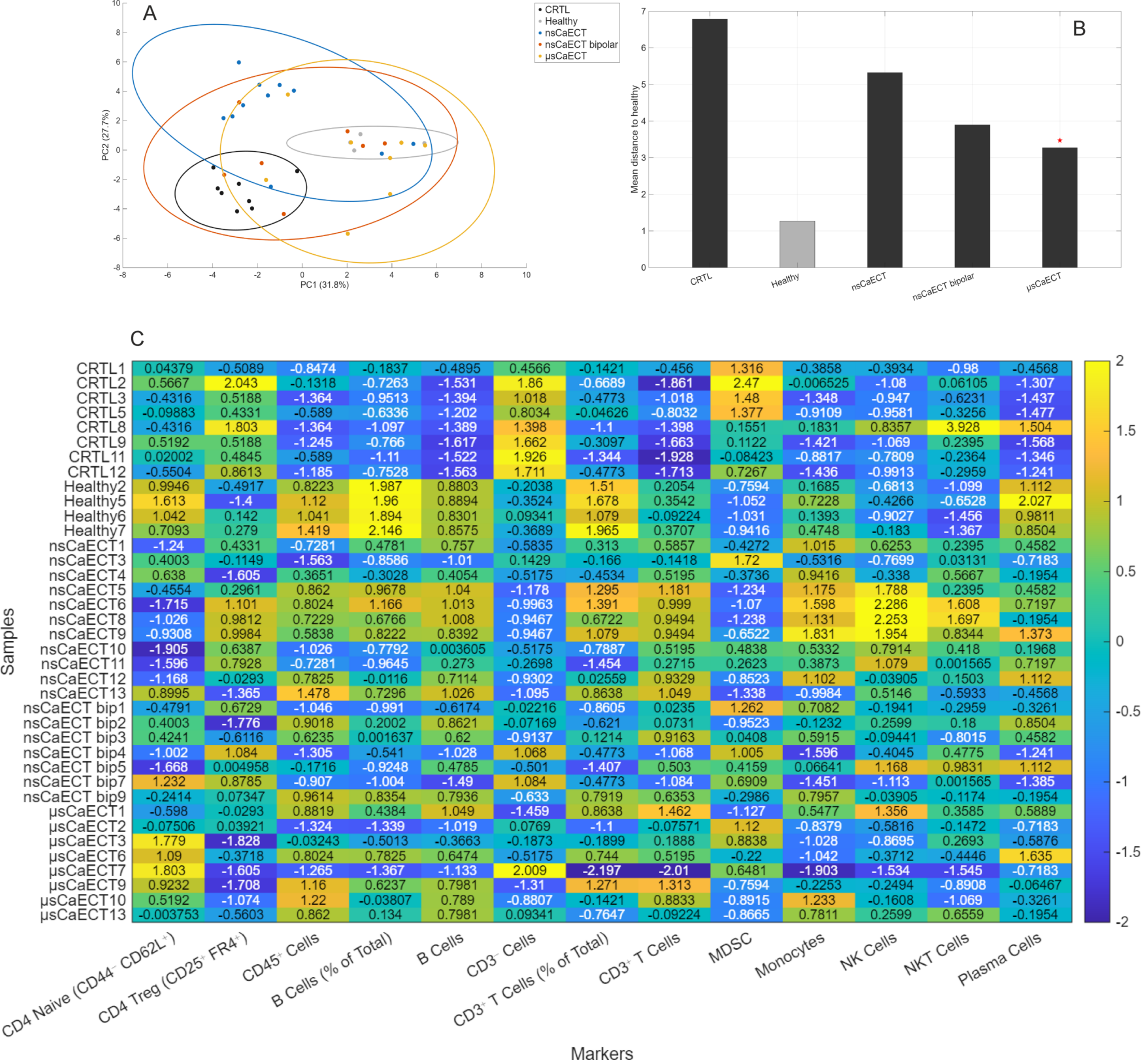
Multivariate analysis of spleen immune cell sub-population composition, where **(A)** PCA shows that untreated tumor-bearing mice (CTRL) cluster distinctly from healthy controls along PC1, reflecting global lymph-node immunosuppression. nsCaECT and μsCaECT groups remain closer to CTRL, whereas the nsCaECT bipolar group shifts toward the healthy cluster, indicating partial normalization. **(B)** Euclidean distance analysis confirms this trend, with nsCaECT bipolar samples showing the smallest mean distance to healthy mice. **(C)** Heat map comparison highlights major most significant (Kruskal–Wallis test, *p* < 0.05) marker differences across groups.

**Figure 5:**
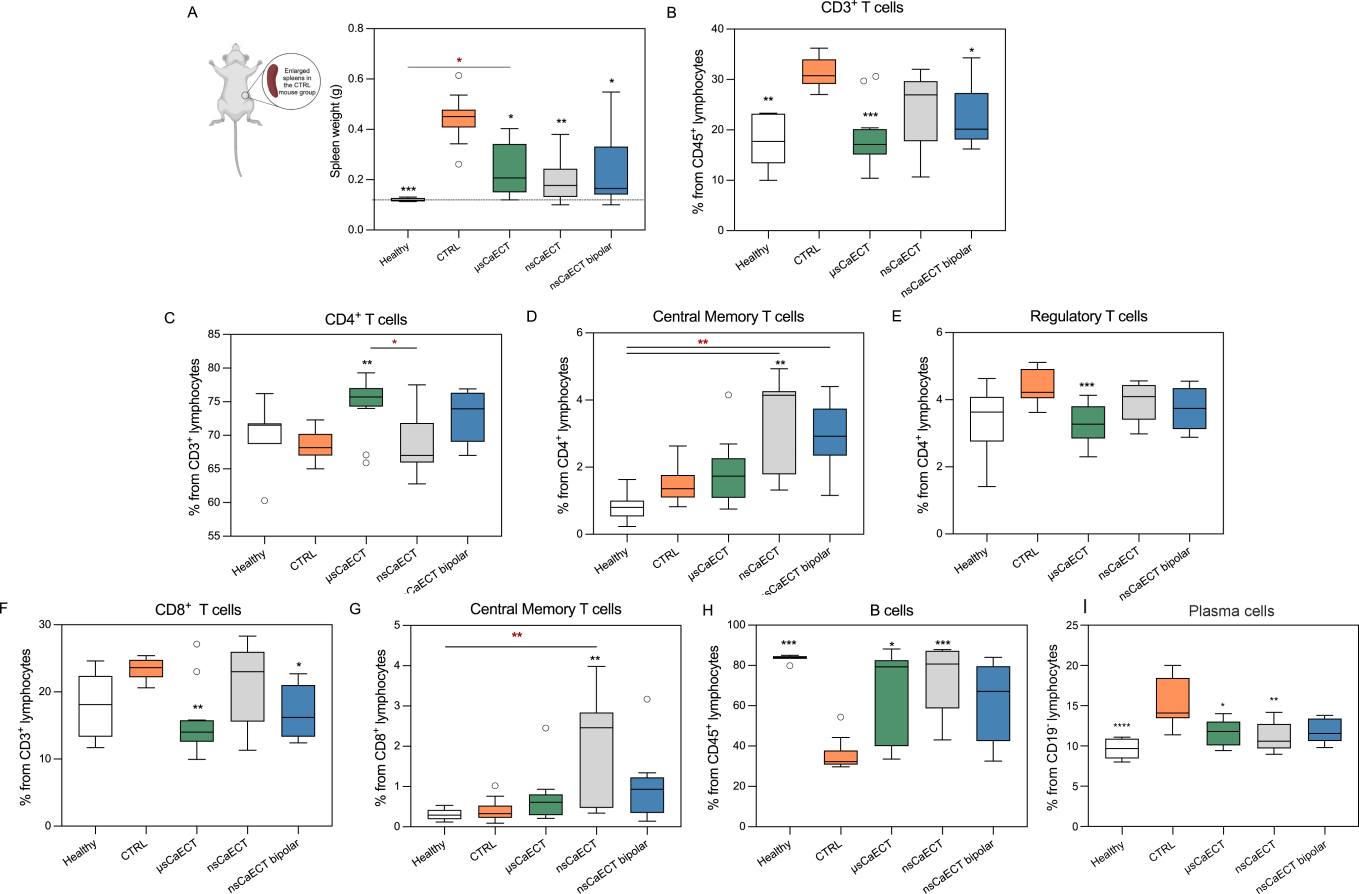
**(A)** Spleen weight in grams; Where healthy refers to the age-matched healthy mice group; B-J) Profiling of lymphoid immune cells after CaECT treatment in spleens. Flow plots represent percentage of T cells **(B)**, CD4^+^ T cells and their subsets **(C–E)**, CD8^+^ T cells and their subsets **(F, G)**, B cells **(H)** and Plasma cells **(I)**. Flow cytometry was performed using BD FACSAria III. Asterisks (*) in black correspond to statistically significant differences compared to the untreated tumor-bearing control (CTRL) and symbols in red (*) correspond to significant differences between treatment groups (Kruskal–Walli’s test with Dunn’s corrected *post hoc* test). Data are presented as Tukey box-and-whisker plots (GraphPad Prism), where the box represents the interquartile range, the line indicates the median, and whiskers extend to 1.5×IQR.

**Figure 6:**
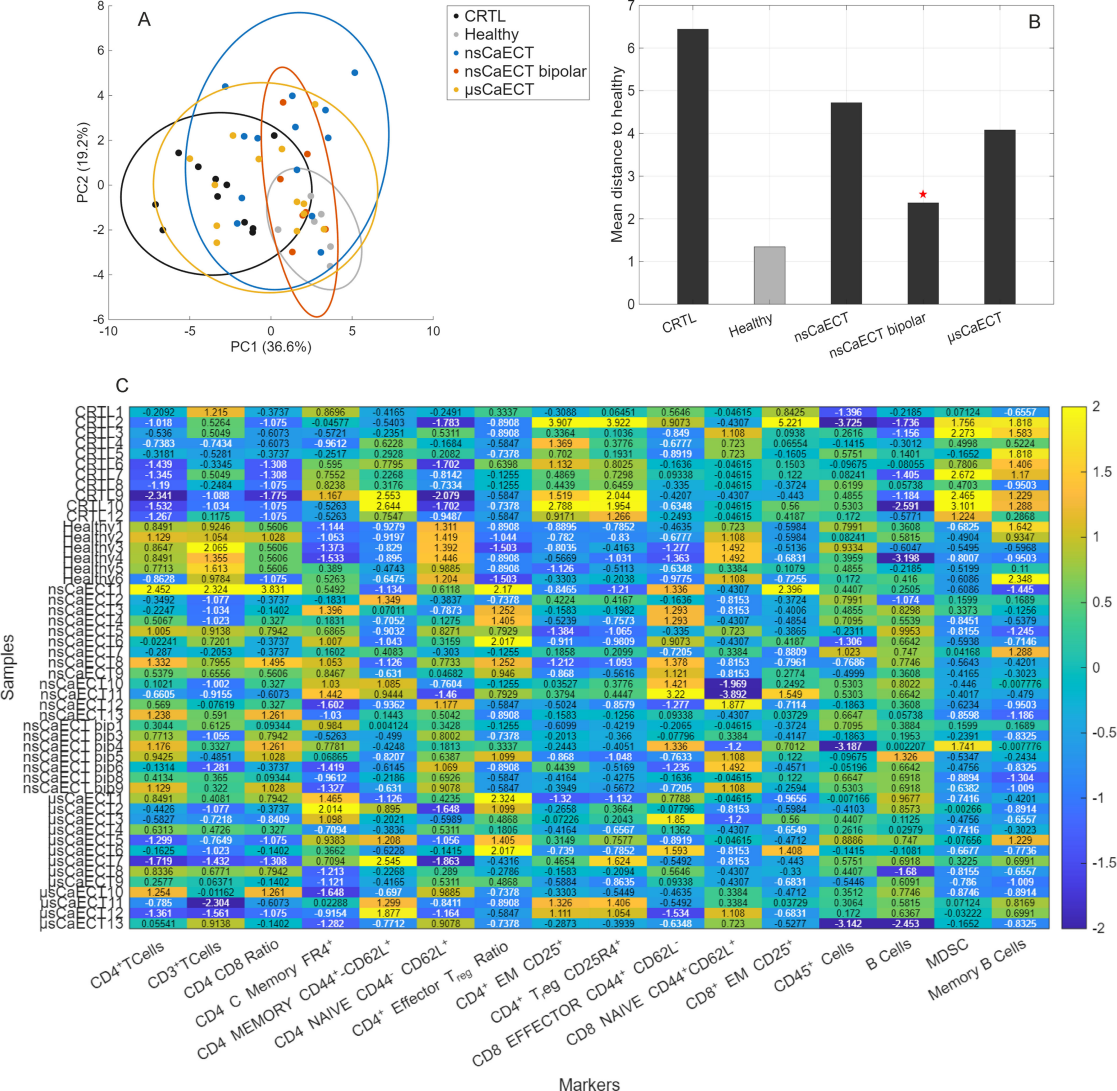
Multivariate analysis of lymph-node immune cell sub-populations composition, where **(A)** PCA shows that untreated tumor-bearing mice (CTRL) cluster distinctly from healthy controls along PC1, reflecting global lymph-node immunosuppression. nsCaECT and μsCaECT groups remain closer to CTRL, whereas the nsCaECT bipolar group shifts toward the healthy cluster, indicating partial normalization. **(B)** Euclidean distance analysis confirms this trend, with nsCaECT bipolar samples showing the smallest mean distance to healthy mice. **(C)** Heat map comparison highlights major marker differences across groups.

**Figure 7:**
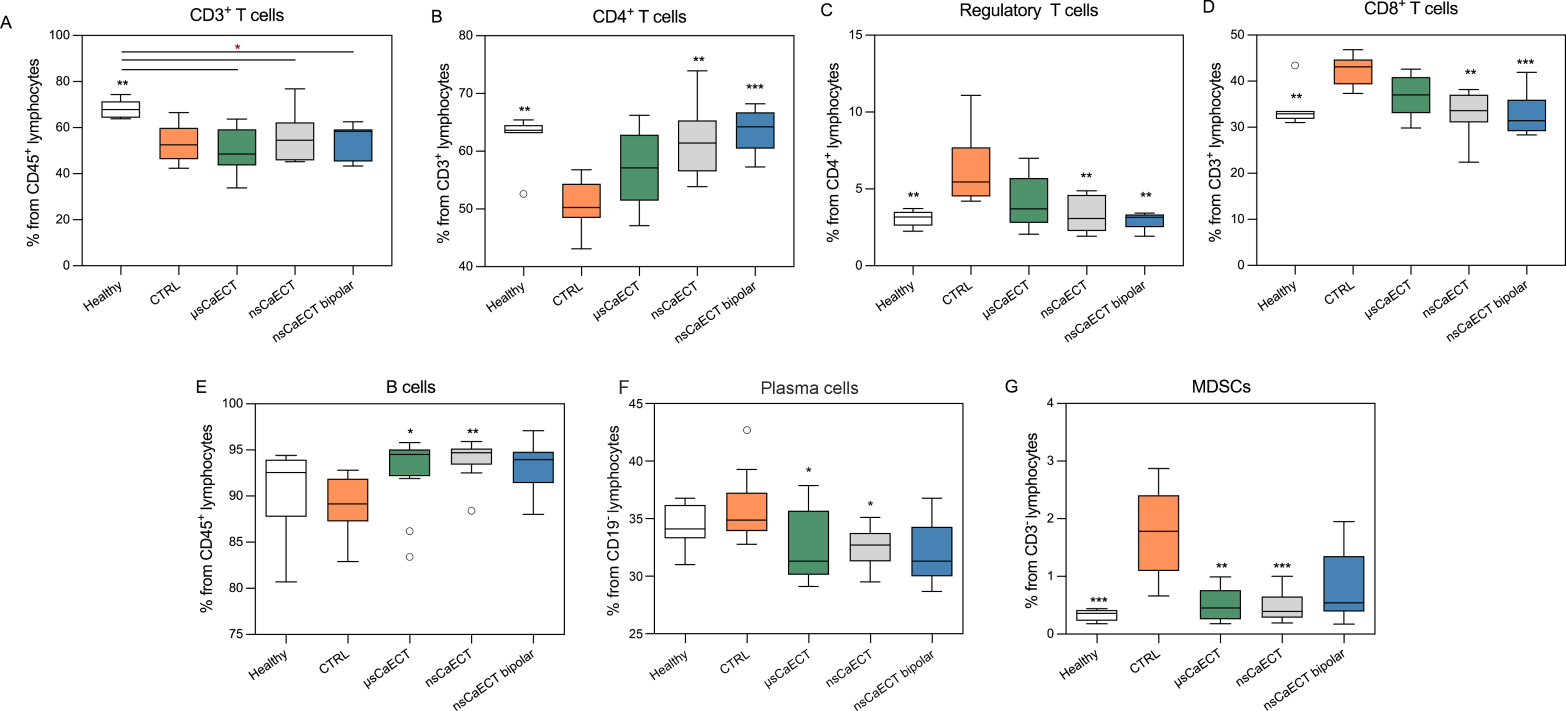
**(A-G)** Profiling of lymphoid and myeloid immune cells after CaECT treatment in tumor-draining lymph nodes. Flow plots represent percentage of T cells **(A)**, CD4^+^ T cells and their subsets **(B, C)**, CD8^+^ T cells **(D)**, B cells **(E)**, Plasma cells **(F)** and myeloid derived suppressor cells (MDSCs; **(G)**. Flow cytometry was performed using BD FACSAria III. Asterisks (*) in black correspond to statistically significant differences compared to the untreated tumor-bearing control (CTRL) and symbols in red (*) correspond to significant differences between treatment groups (Kruskal–Walli’s test with Dunn’s corrected *post hoc* test). Data are presented as Tukey box-and-whisker plots (GraphPad Prism), where the box represents the interquartile range, the line indicates the median, and whiskers extend to 1.5×IQR.

At the end of the experiment (day 20), the mice were sacrificed, and spleens (**Figures 4**, **5**), tumor-draining lymph nodes (LNs) (**Figures 6**, **7**), and blood (**Supplementary Figure 4**) were collected. Single-cell suspensions from the spleens and LNs were processed using flow cytometry to analyze the immune cells. For comprehensive multivariate analysis of spleen immune cell sub-populations composition, we firstly evaluated PCA analysis along with heat map of most statistically significant (Kruskal–Wallis test, p < 0.05) marker markers as it shown in **Figure 4**.

PCA of spleen immune markers revealed treatment-dependent differences in global immune profiles (**Figure 4**). The first two principal components explained a substantial proportion of the total variance (PC1: 31.8%, PC2: 27.7%). Healthy samples formed a compact cluster, whereas CTRL tumor-bearing mice were clearly separated from healthy controls in PCA space. All treatment groups occupied intermediate positions between CTRL and healthy samples, with distinct clustering patterns. Quantification of multivariate deviation using Euclidean distance to the healthy centroid in PC1–PC2 space showed the greatest distance in CTRL animals, reduced distances in all treatment groups, and the smallest mean distance in the μsCaECT group (**Figure 4B**).

A heatmap of z-scored spleen immune markers showing group-dependent differences demonstrated coordinated, marker-specific immune alterations across experimental conditions (**Figure 4C**). CTRL samples exhibited pronounced deviations across multiple T-cell–associated markers, including total CD3^+^ and CD4^+^ T cells, CD4/CD8 ratio, and CD4^+^ and CD8^+^ effector, memory, and naïve subsets, as well as altered CD4 effector–to–Treg ratios. Clear and consistent differences were also evident in B-cell–associated populations, including total B cells, memory B cells, and plasma cells, which showed distinct expression patterns across experimental groups. In contrast, NK-cell–associated markers displayed less pronounced and more variable changes across treatment conditions. Treated groups generally exhibited intermediate profiles between CTRL and healthy samples, with μsCaECT showing immune patterns more closely resembling healthy controls for several adaptive immune markers, whereas nsCaECT and nsCaECT bipolar groups demonstrated greater heterogeneity across both adaptive and innate immune compartments.

Based on these global, multivariate immune differences between experimental groups, we next examined spleen size and individual immune cell sub-populations to determine how these immune system changes relate to splenomegaly and specific cellular changes. First, we noticed enlarged spleens or splenomegaly in the control group of tumor-bearing mice. These differences became more pronounced when significant changes in spleen weight were observed (**Figure 5A**). All treatment groups, as well as the healthy mice group, had significantly lower spleen weights than the control group. Moreover, the spleen weight in the treatment groups was similar to that of healthy mice, which can be explained by the positive treatment outcome following ECT.

As shown in **Figure 5B**, CD3^+^ cells were distinguished and showed a decrease compared to that in the CTRL group, consistent with the percentages of T cells in healthy mice. A significant increase in the percentage of CD4^+^ T cells was observed only in microsecond calcium ECT-treated mice compared with untreated (CTRL) mice. At the same time, in the same treatment group (μsCaECT), the percentage of CD8^+^ T cells decreased. Moreover, the percentages of CD4^+^ and CD8^+^ central memory cells (T_CM_; CD44^+^CD62L^+^) were significantly higher in nsCaECT-treated mice than in untreated and healthy mice (**Figures 4D, G**). Additionally, a subset of regulatory CD4^+^ T cells (T_reg_; CD4^+^CD25^+^FR4^+^) was analyzed. It is apparent (**Figure 5E**) that the percentage in all treatment groups was similar to that in healthy mice, but only after μsCaECT treatment was the percentage of CD4^+^ T_reg_ cells significantly lower than that in untreated tumor-bearing mice.

The percentage of splenic B cells (CD3^-^CD19^+^) increased in all treatment groups compared to that in control mice, with values matching those of healthy mice (**Figure 5H**). However, no differences were observed among the different treatment groups of plasma cells (PCs; CD19^+^CD138^+^). Additionally, circulating blood was collected to determine the specific antitumor IgG antibodies, and no significant differences were found; the MFI values remained very similar (data not shown; see S4 for details).

We also investigated the effect of treatment on the changes in tumor-draining lymph nodes. We first performed PCA analysis as it showed in **Figure 6**. PCA of lymph nodes immune markers showed treatment-dependent differences in global immune profiles (**Figure 6**). The first two principal components explained a substantial proportion of the total variance (PC1: 36.6%, PC2: 19.2%).

PCA (**Figure 6A**) showed that untreated tumor-bearing mice (CTRL) formed a compact cluster distinctly separated from healthy controls along PC1, consistent with a globally immunosuppressed lymph-node phenotype. Both nsCaECT and μsCaECT groups remained positioned close to the CTRL cluster, whereas the nsCaECT bipolar group shifted toward the healthy profile and exhibited the smallest Euclidean distance to healthy mice, indicating partial normalization of lymph-node immunity (**Figure 6B**). Heat-map analysis (**Figure 6C**) supported these findings: CTRL mice showed reduced CD4/CD8 ratios, lower NKT-cell and plasma-cell frequencies, and elevated MDSCs relative to healthy animals, reflecting tumor-associated immune dysfunction. In contrast, nsCaECT bipolar treatment consistently reduced MDSC abundance and increased B cells, NKT cells, plasma cells, and several CD4^+^ and CD8^+^ activation-associated subsets, with several values approaching or exceeding healthy levels. Other treatment groups showed more heterogeneous and less pronounced changes.

Collectively, these results demonstrate that nsCaECT bipolar induces the strongest shift toward an immunoactivity, less suppressive lymph-node phenotype, partially reversing immune alterations observed in untreated tumor-bearing mice. Markers associated with key lymphocyte populations, including CD4^+^ T cells, CD8^+^ T cells, and CD3^+^ T cells, were among those with the strongest significance, suggesting that T cell subsets are highly responsive to the experimental conditions. Additionally, several functional and differentiation markers, such as CD4^+^ effector CD44^+^CD62L^-^, Naïve CD4^+^ CD44^-^CD62L^+^ T cells, and CD8^+^ memory CD44^+^CD62L^+^, indicate alterations in activation and memory status. The inclusion of innate-like populations such as NKT cells and B cells highlights a broader immune modulation beyond classical T cell responses.

It was observed (**Figure 7A**) that the percentage of T cells (CD3^+^) in the tumor-draining lymph nodes of treated mice were at the same level as that in the CTRL group but significantly lower than that in healthy mice. A significantly increased percentage of CD4^+^ T cells, normalized to healthy mice, was observed after sub-microsecond unipolar and bipolar calcium ECT. Moreover, a decrease in the CD4^+^ regulatory T cell subset, more pronounced than that in splenic T_regs_, was observed after treatment compared to that in the tumor-bearing control (**Figure 7C**). CD4^+^ T cells and T_reg_ cells exhibited the opposite patterns. In the group with the lowest CD4^+^ T cell percentage, the T_reg_ percentage was the highest and vice versa. Furthermore, CD8^+^ T cells decreased in the nanosecond calcium ECT-treated group compared to that in CTRL mice, similar to healthy mice.

**Figure 7E** shows a significant increase in B cell percentage in lymph nodes after both microsecond and nanosecond CaECT compared to that in untreated mice. The percentage of plasma cells was significantly decreased in the treatment groups compared to that in the control (CTRL) mice; however, it appears that the percentage has normalized and is now very similar to that in healthy mice. A significant decrease in myeloid-derived suppressor cells (MDSCs; CD11b^+^Gr1^+^) was observed in all calcium ECT treatment groups, comparable to that in healthy mice.

The control group exhibited immune dysregulation, including spleen enlargement, altered T-cell and B-cell populations, and reduced cytotoxic and memory responses. μsECT and nsECT generally reduced spleen enlargement, normalized immune cell populations, and enhanced central memory T cell responses. Interestingly, nsECT bipolar treatment was the most effective in promoting central memory T cells, suggesting long-term immune benefits. Our findings imply that the EP protocols used can modulate both cellular and humoral adaptive immune responses, potentially mitigating the pathological effects observed in the CTRL group. Finally, the selection of an appropriate protocol depends on expected therapeutic outcomes. nsECT bipolar protocols seem preferable for adaptive immune enhancement, whereas nsECT protocols might be better for addressing inflammation and cytotoxic immunity.

## 4 Discussion

In this study, we aimed to characterize CaECT using both unipolar and bipolar sub-microsecond pulses in the 4T1 tumor model. For the first time, we directly compared the immunogenic response elicited by bipolar sub-microsecond pulses with the standard microsecond protocol (ESOPE) and high-frequency unipolar sub-microsecond bursts. Our results demonstrate that unipolar pulses, both in the microsecond and sub-microsecond range, could induce partial or complete tumor regression and eliciting a systemic immune response, consistent with previous observations (30, 64). Notably, protocols employing bipolar pulses achieved comparable outcomes, with complete tumor response equivalent to that observed with unipolar pulses, which is an unexpected result.

Membrane charging dynamics may also contribute to the observed differences between *in vitro* and *in vivo* responses. According to the Schwan equation, the induced transmembrane potential (TMP) depends on both the applied electric field and the membrane charging time constant (65). Therefore, nanosecond pulses may terminate before the membrane reaches steady-state TMP, unlike conventional microsecond protocols. However, this limitation can be compensated by higher electric field amplitudes, while the capacitive nature of the membrane and high pulse repetition rates can enhance cumulative membrane charging (66, 67), supporting effective electroporation under the applied conditions.

Both the *in vitro* data and previous pilot studies indicated that biphasic sequences (e.g., 5 kV/cm, 500 + 500 ns, 100 pulses at 1 MHz) trigger hindered efficacy of ECT (68). However, electroporation-based therapies are highly sensitive to multiple parameters, amplitudes, phase ratio (69), inter phase delay (70, 71), pulse width and waveform asymmetry (55); thus, these results cannot be directly extrapolated to other high-frequency bipolar protocols. In the present study, different tumor model, higher pulse amplitudes and higher number of pulses were used. The hypothesis that bipolar cancellation can be overcome by increasing the pulse amplitude and the pulse number *in vivo* requires further investigation. Currently it’s not possible to form conclusions on why the *in vitro* effects are not transferrable to *in vivo*, which might be the consequence of conductivity differences and a more complex structure of tissue when compared to suspension, which could have altered the polarization dynamics. In heterogeneous tissues, current follow paths of least resistance shaped by conductivity differences, extracellular matrix composition, and vascularization (51, 72), resulting in spatially variable electric-field distributions, particularly when tumor and surrounding tissues differ in conductivity. Moreover, due to tumor tissues structural heterogeneity, local redistribution of the applied electric field during treatment is expected (63, 73–75). Variations in cell density, necrotic regions, and vascular structures can result in spatial differences in electrical conductivity, causing certain tumor regions to experience slightly different electric field strengths, which can also affect the tumor regrowth.

Additionally, unipolar protocols are associated with stronger polarization at electrode–tissue interfaces (76, 77). This effect can be further amplified by intratumoral injection of highly conductive calcium, which may shunt current through fluid-rich regions and away from dense tumor cell clusters. In contrast, bipolar pulses confine the field between electrodes and promote more symmetric and homogeneous exposure (78). Although bipolar pulses generally require higher total energy to achieve comparable cellular permeabilization due to phase cancellation, their rapid polarity alternation mitigates impedance mismatches, smooths field inhomogeneities (79), and limits hotspots near electrode edges.

Calcium-induced regulated cell death and immunogenic signaling can be triggered at relatively low permeabilization thresholds (59), potentially mediated through mitochondrial and ER stress (80–82). Calcium loading within tumor tissue can subtly influence local electric field distribution because Ca²^+^ ions alter the effective conductivity and permittivity of the intracellular and interstitial compartments (83). As a result, tumors with substantial intracellular or extracellular calcium may exhibit local reductions in voltage drop, smoother field gradients, and a tendency for the electric field lines to redistribute toward these more conductive zones. While the magnitude of this effect is modest compared with macroscopic factors (geometry, hydration, fibrosis), it may still contribute to more homogeneous field penetration during CaECT.

One of the major goals of this study was to assess the immunomodulatory effects of bipolar pulse therapy. In a previous study (on unipolar sequences), we demonstrated a statistically significant increase in spleen weight in untreated CTRL mice, whereas after successful CaECT treatment, the mice had normal weight and sized spleens, similar to those of healthy mice (54, 64), which was repeatable and confirmed in this study too. Splenomegaly is associated with tumor progression (84), which is also the case in our murine experiment, in which only untreated tumor-bearing mice were found to have enlarged spleens. Additionally, Guo et al. reported splenomegaly in the context of nano-pulse stimulation (NPS) in a 4T1-luc breast cancer model (31).

Regarding immunomodulatory effects, it is well known that the activation and expansion of CD4^+^, especially CD8^+^, immune cells are crucial for an effective antitumor immune response (85). In our study, we detected an increased splenic CD4^+^ T cell percentage but decreased CD8^+^ T cell CaECT treatment compared to untreated tumor-bearing mice. The increase in CD4^+^ was more pronounced using sub-microsecond pulses, which we speculate is attributed to a more homogeneous electric field distribution within the tumor owing to the higher frequency component of the bursts. However, the increase in CD8^+^ T cells did not always show an effective antitumor response, as shown by the data from untreated tumor-bearing mice (**Figures 4C, F**, **5B, D**), where suppressor cells increased, and no recovery occurred (**Figure 3B**). Suppressor chains should be considered when evaluating CD4^+^ and CD8^+^ T cell data.

B cells are crucial for adaptive immunity through antigen presentation and antibody production, although their role in tumor immunity varies across different tumor types (86). A significant increase in B cell percentage was observed in the spleens and lymph nodes after unipolar microsecond and nanosecond CaECT, with values matching those of the healthy mice (**Figures 4H**, **5E**). This was not observed for bipolar pulses, where B cell percentages showed an increasing trend but did not reach statistical significance. Additionally, a decrease in the percentage of plasma cells in the lymph nodes was observed (similar to healthy mice) following CaECT regardless of the protocol. No changes in circulating antitumor antibodies in the blood were observed (**Supplementary Figure 4**).

The immune patterns observed in lymph nodes and spleen are consistent with recent studies showing that effective local tumor eradiation can reshape immune responses beyond the tumor site (87). Prior work on electroporation-based and other focal therapies indicates that relief of tumor-associated immunosuppression in draining lymph nodes – often reflected by reduced suppressive myeloid and regulatory T-cell populations and increased activated T cells – is important for downstream antitumor immunity (88–90). Accordingly, the coordinated modulation of CD4^+^ and CD8^+^ differentiation markers and normalization of CD4/CD8 ratios observed here are consistent with improved conditions for antigen-specific T-cell priming rather than nonspecific immune activation (91). At the systemic level, modulation of splenic B-cell and plasmacell sub-populations align with growing evidence that humoral and adaptive immune compartments can reflect sustained immune engagement after immunogenic tumor cell death, even when innate effector populations such as NK cells show limited change. Together, these findings support emerging models in which treatment modality and electric-field configuration influence not only local tumor control but also compartment-specific immune regulation, which may contribute to differences in therapeutic efficacy and inform optimization of electroporation parameters to enhance long-term immune observation.

It has been reported that in antitumor immunity, T_CM_ plays a crucial role by producing higher levels of cytokines and exhibiting stronger cytotoxic activity *in vitro* (92, 93). Thus, changes in the splenic central memory T cell subset were observed, with a significant increase in CD4^+^ and CD8^+^ T_CM_ cells when unipolar nanosecond pulses were used compared to untreated and healthy mice (**Figures 4D, G**). This was not observed for bipolar pulses, where the average increase was not significant, highlighting the differences between unipolar and bipolar bursts, despite bipolar pulses requiring double the energy. The increase in CD8^+^ central memory T cells agree with previous studies focusing on nanosecond pulses (30, 31, 94). However, functional validation of systemic anti-tumor immune memory would require tumor re-challenge experiments, which were beyond the scope of the present study.

Myeloid-derived suppressor cells (MDSCs) and regulatory T (T_reg_) cells are key components of the cancer immunosuppressive network. A decrease in CD4^+^ T_reg_ in spleens and tumor-draining lymph nodes was observed after treatment with all CaECT protocols, compared to the tumor-bearing control (**Figures 4E**, **5C**), and the percentage was similar to that in healthy mice. CD4^+^ and T_reg_ cells exhibited opposite patterns. In the group with the lowest CD4^+^ percentage, T_reg_ percentage was the highest, and vice versa. A statistically significant reduction in MDSCs, comparable to that in healthy mice, was observed in all calcium ECT treatment groups compared to that in the untreated controls (Figure. 5G). An increase in T_CM_ and a decrease in suppressor cells suggest that CaECT affects the anti-cancer immune response and cancer immunotolerance. Data from fully recovered mice in the treatment groups support these assumptions. Other studies (28, 30, 31, 64, 94) (95) have reported a decrease in T_reg_ cells and MDSCs after NPS, IRE, ECT, and CaECT treatment, which agrees with our data. Recent studies have shown that MDSCs can influence the *de novo* development and induction of T_reg_ cells from naive T cell (90). The causal interplay between MDSCs and Tregs under treatment remains unclear. While MDSCs can promote the *de novo* development and induction of Tregs from naïve T cells, Tregs can, in turn, induce and amplify MDSCs through soluble factors and cell contact (96). Does the electric field-based therapies in this bidirectional relationship primarily disrupt MDSCs, thereby removing a key source of Treg induction, or does it directly target Tregs, withdrawing the signals necessary for MDSC maintenance remains to be established. Clarification of this interplay of effects will be needed for optimizing immunomodulatory ablation strategies (97).

Our results support the positive immunomodulatory activity of CaECT. However, bipolar high-frequency nanosecond pulses show no clear advantage over unipolar sub-microsecond pulses in terms of primary tumor response or immunomodulatory effects. Unipolar nanosecond pulses improve muscle contractions (compared to ESOPE), enhance electric field homogeneity owing to their higher frequency, and yield a stronger antitumor response. In contrast, bipolar pulses, which may trigger bipolar cancellation and require double the energy, appear to be unjustified.

## 5 Conclusions

In summary, our results demonstrate that both microsecond and sub-microsecond range uni- and bipolar pulses can be used in calcium-based ECT, leading to partial or complete tumor treatment. Additionally, we showed that CaECT treatment induces a systemic immune response by increasing the percentage of splenic central memory T cells, suppressing cancer immune tolerance by decreasing CD4^+^ regulatory T cells in both the spleen and tumor-draining lymph nodes, and reducing MDSCs in the lymph nodes. The applicability of bipolar protocols for CaECT is still debatable because of the lack of objective benefits in terms of primary tumor growth suppression and immunomodulatory effects compared to unipolar procedures. The reason why bipolar cancellation was not detected with 7 kV/cm × ↑300 + ↓300 ns × 250 (1 MHz), despite its presence *in vitro*, is unknown and requires further research.

## Data Availability

The datasets presented in this study can be found in online repositories. The names of the repository/repositories and accession number(s) can be found in the article/**Supplementary Material**.
