# Supplementary material for: Evaluation of unipolar and bipolar nanosecond pulses for calcium electrochemotherapy and immune response
Source: Front Immunol. 2026 Apr 20;17:1805130. doi: 10.3389/fimmu.2026.1805130 (PMC13136128; doi:10.3389/fimmu.2026.1805130)
Supplement: Supplementary file 1 [file DataSheet1.docx]

Supplementary Material

**Bipolar Nanosecond-Pulses for Electrochemotherapy: Cancellation Phenomenon and Limited Benefits for Immunomodulation**

Eivina Radzevičiūtė-Valčiukė^1,2^, Augustinas Želvys^1,2^, Veronika Malyško^1,2*^, Eglė Mickevičiūtė-Zinkuvienė^1,2^, Paulina Malakauskaitė^1,2^, Barbora Lekešytė^1,2^, Jovita Gečaitė^1^, Auksė Zinkevičienė^1^, Vytautas Kašėta^1^, Julita Kulbacka^1,3^, Joanna Rossowska^4^, and Vitalij Novickij^1,2*^

^1^State Research Institute Centre for Innovative Medicine, Department of Immunology and Bioelectrochemistry, Vilnius, Lithuania

^2^Faculty of Electronics, Vilnius Gediminas Technical University, Vilnius, Lithuania

^3^Wroclaw Medical University, Faculty of Pharmacy, Department of Molecular and Cellular Biology, Wroclaw, Poland

^4^ Hirszfeld Institute of Immunology and Experimental Therapy, Polish Academy of Sciences, Wroclaw, Poland

*** Correspondence:**dr. Veronika Malyško

v.malysko@vilniustech.lt

Prof. dr. Vitalij Novickij
vitalij.novickij@vilniustech.lt

**Supplementary Table 1.** List of antibodies used. Listed are antibody name, dilutions, manufacturer and ID product codes are presented.

| Anti-mouse antibodies against cell markers: | Manufacturer | Identifier | Dilution |
| --- | --- | --- | --- |
| PerCP Anti-CD45 | BD | 2329575 | 1:200 |
| FITC Anti-CD44 | BD | 553133 | 1:400 |
| AF700 Anti-CD8 | Invitrogen | 2075802 | 1:400 |
| AF674 Anti-CD25 | Invitrogen | 2029795 | 1:200 |
| BV510 Anti-CD3 | Biolegend | 100234 | 1:200 |
| BV421 Anti-FR4 | BD | 744119 | 1:400 |
| PE/Cy7 Anti-CD62L | Biolegend | 104417 | 1:400 |
| PE Anti-CD4 | MACS | 5111206117 | 1:800 |
| FITC Anti-CD3 | BD | 2159105 | 1:800 |
| APC Cy7 Anti-CD49b | Molecular probes | D2213-R306X | 1:400 |
| APC Anti-CD138 | Invitrogen | UJ2867901A | 1:200 |
| SuperBright 600™ Anti-CD27 | Invitrogen | 2212412 | 1:400 |
| eFluor™ 506 Anti-CD19 | Invitrogen | 2107357 | 1:800 |
| Pe-TxRed Anti-CD11b | Invitrogen | 2159105 | 1:800 |
| PE Anti-Gr1 | Biolegend | 108407 | 1:200 |
| AF488 Anti-mouse IgG | Invitrogen | 2379467 | 1:200 |

Anti-mouse FcγR (Fc block) – supernatant from hybridoma cells secreting monoclonal antibodies against Fc receptor (home-made, CIM, Lithuania).

Fluorescent stains LIVE/DEAD™ Fixable Near-IR Stain (Thermo Fisher Scientific, USA; 2339909).

**Supplementary Figure 1.** A common gating strategy to distinguish singlets, live cells, and immune cells


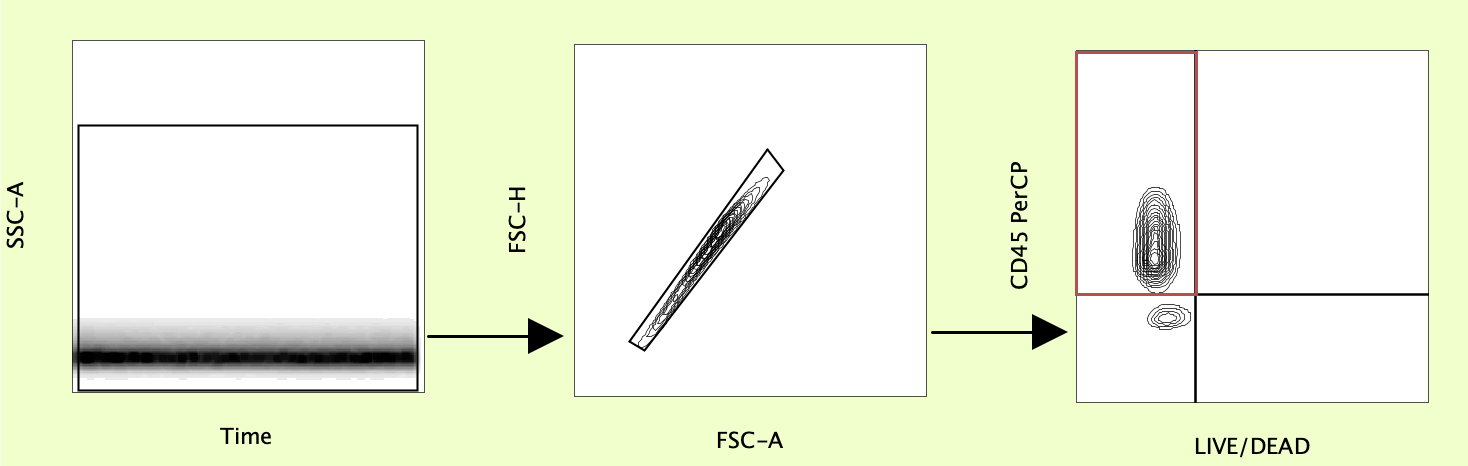


R1 gate

**Supplementary Figure 2.** A common gating strategy to distinguish T cell subsets


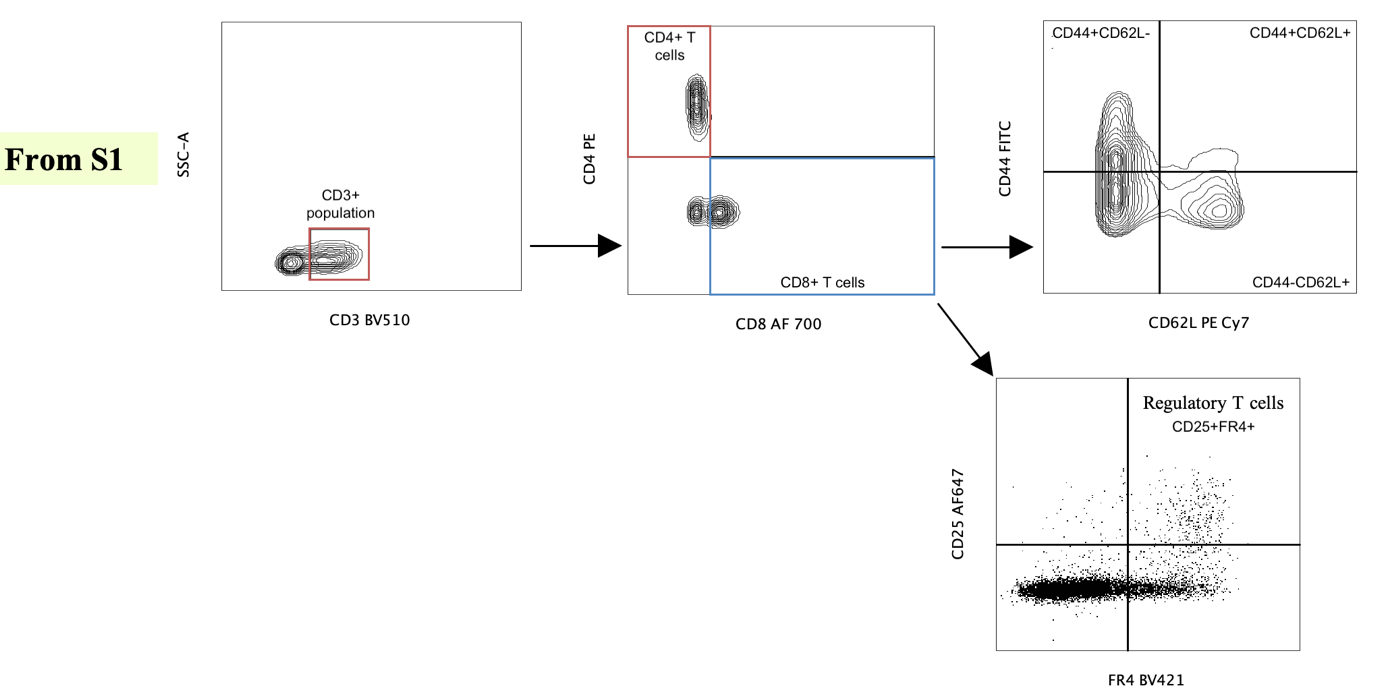


R1 gate

**Supplementary Figure 3.** A common gating strategy to distinguish Myeloid and B cell subsets.


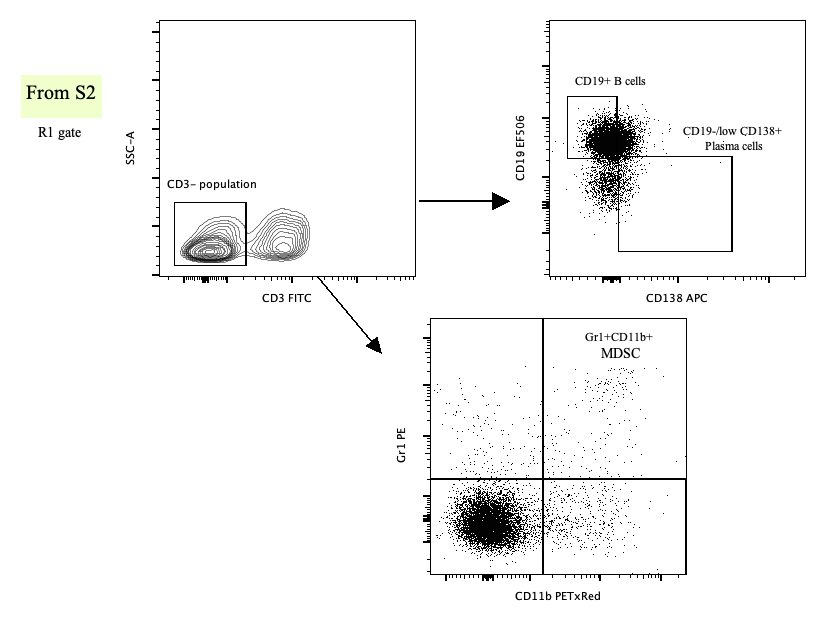


**Supplementary Figure 4.** The median fluorescence intensity (MFI) of antitumor IgG antibodies. Flow cytometry was performed using Amnis FlowSight. Data are presented in Tukey box plots; the upper and lower whiskers represent scores outside the middle 50%.


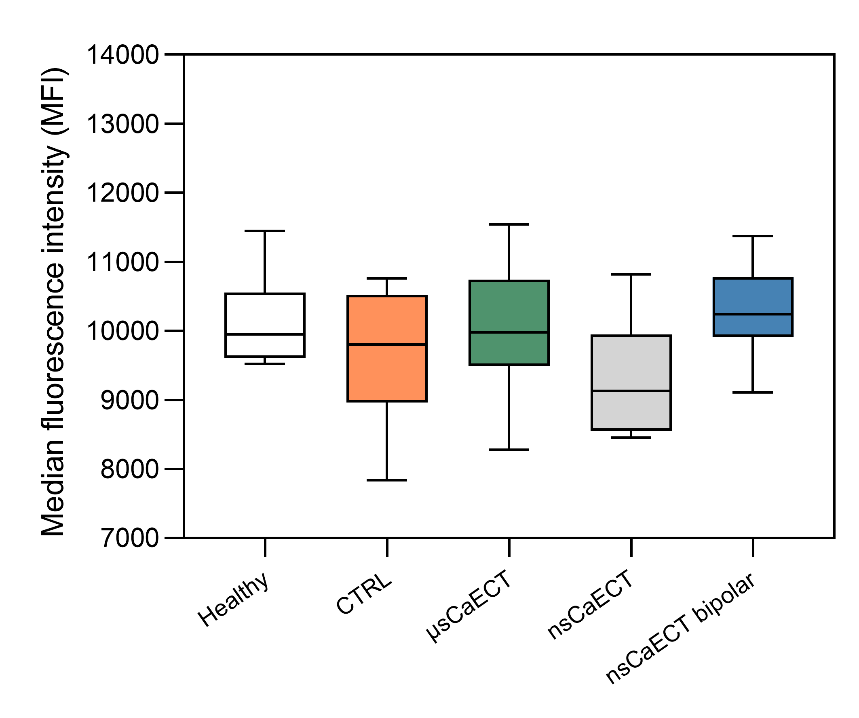


**Supplementary Table 2.** List of p-values for reported changes in immune cell populations.

| Number of families | 1 |  |
| --- | --- | --- |
| Number of comparisons per family | 10 |  |
| Alpha | 0.05 |  |
|  |  |  |
|  |  |  |
| From spleens |  |  |
|  |  |  |
| CD3^+^ cells |  |  |
| Dunn's multiple comparisons test | Mean rank diff. | Adjusted P Value |
| Healthy vs. CTRL | -29.29 | 0.002 |
| Healthy vs. μsCaECT | -2.064 | >0.9999 |
| Healthy vs. nsCaECT | -14.26 | 0.663 |
| Healthy vs. nsCaECT bipolar | -10.23 | >0.9999 |
| CTRL vs. μsCaECT | 27.23 | 0.0002 |
| CTRL vs. nsCaECT | 15.04 | 0.1695 |
| CTRL vs. nsCaECT bipolar | 19.06 | 0.0466 |
| μsCaECT vs. nsCaECT | -12.19 | 0.4814 |
| μsCaECT vs. nsCaECT bipolar | -8.169 | >0.9999 |
| nsCaECT vs. nsCaECT bipolar | 4.023 | >0.9999 |
|  |  |  |
| CD4^+^ cells |  |  |
| Dunn's multiple comparisons test | Mean rank diff. | Adjusted P Value |
| Healthy vs. CTRL | 8.19 | >0.9999 |
| Healthy vs. μsCaECT | -13.37 | 0.7493 |
| Healthy vs. nsCaECT | 6.78 | >0.9999 |
| Healthy vs. nsCaECT bipolar | -7.543 | >0.9999 |
| CTRL vs. μsCaECT | -21.56 | 0.0077 |
| CTRL vs. nsCaECT | -1.41 | >0.9999 |
| CTRL vs. nsCaECT bipolar | -15.73 | 0.2179 |
| μsCaECT vs. nsCaECT | 20.15 | 0.0134 |
| μsCaECT vs. nsCaECT bipolar | 5.831 | >0.9999 |
| nsCaECT vs. nsCaECT bipolar | -14.32 | 0.3352 |
|  |  |  |
| TCM from CD4^+^ cells |  |  |
| Dunn's multiple comparisons test | Mean rank diff. | Adjusted P Value |
| Healthy vs. CTRL | -12.51 | 0.9445 |
| Healthy vs. μsCaECT | -16.43 | 0.259 |
| Healthy vs. nsCaECT | -33.31 | <0.0001 |
| Healthy vs. nsCaECT bipolar | -31.04 | 0.0009 |
| CTRL vs. μsCaECT | -3.917 | >0.9999 |
| CTRL vs. nsCaECT | -20.8 | 0.0096 |
| CTRL vs. nsCaECT bipolar | -18.53 | 0.0756 |
| μsCaECT vs. nsCaECT | -16.88 | 0.0621 |
| μsCaECT vs. nsCaECT bipolar | -14.61 | 0.322 |
| nsCaECT vs. nsCaECT bipolar | 2.274 | >0.9999 |
|  |  |  |
|  |  |  |
| Treg cells |  |  |
| Dunn's multiple comparisons test | Mean rank diff. | Adjusted P Value |
| Healthy vs. CTRL | -18.79 | 0.1364 |
| Healthy vs. μsCaECT | 6.731 | >0.9999 |
| Healthy vs. nsCaECT | -9.423 | >0.9999 |
| Healthy vs. nsCaECT bipolar | -4.2 | >0.9999 |
| CTRL vs. μsCaECT | 25.52 | 0.0007 |
| CTRL vs. nsCaECT | 9.369 | >0.9999 |
| CTRL vs. nsCaECT bipolar | 14.59 | 0.3338 |
| μsCaECT vs. nsCaECT | -16.15 | 0.1014 |
| μsCaECT vs. nsCaECT bipolar | -10.93 | >0.9999 |
| nsCaECT vs. nsCaECT bipolar | 5.223 | >0.9999 |
|  |  |  |
| CD8^+^ cells |  |  |
| Dunn's multiple comparisons test | Mean rank diff. | Adjusted P Value |
| Healthy vs. CTRL | -16.72 | 0.2819 |
| Healthy vs. μsCaECT | 6.61 | >0.9999 |
| Healthy vs. nsCaECT | -10.43 | >0.9999 |
| Healthy vs. nsCaECT bipolar | 3.421 | >0.9999 |
| CTRL vs. μsCaECT | 23.33 | 0.0027 |
| CTRL vs. nsCaECT | 6.292 | >0.9999 |
| CTRL vs. nsCaECT bipolar | 20.14 | 0.0332 |
| μsCaECT vs. nsCaECT | -17.04 | 0.0669 |
| μsCaECT vs. nsCaECT bipolar | -3.188 | >0.9999 |
| nsCaECT vs. nsCaECT bipolar | 13.85 | 0.3983 |
|  |  |  |
| TCM from CD8^+^ |  |  |
| Dunn's multiple comparisons test | Mean rank diff. | Adjusted P Value |
| Healthy vs. CTRL | -3.375 | >0.9999 |
| Healthy vs. μsCaECT | -12.08 | >0.9999 |
| Healthy vs. nsCaECT | -24.77 | 0.0078 |
| Healthy vs. nsCaECT bipolar | -17.28 | 0.2928 |
| CTRL vs. μsCaECT | -8.702 | >0.9999 |
| CTRL vs. nsCaECT | -21.39 | 0.0068 |
| CTRL vs. nsCaECT bipolar | -13.9 | 0.4502 |
| μsCaECT vs. nsCaECT | -12.69 | 0.3966 |
| μsCaECT vs. nsCaECT bipolar | -5.201 | >0.9999 |
| nsCaECT vs. nsCaECT bipolar | 7.491 | >0.9999 |
|  |  |  |
| B cells |  |  |
| Dunn's multiple comparisons test | Mean rank diff. | Adjusted P Value |
| Healthy vs. CTRL | 35.73 | <0.0001 |
| Healthy vs. µsCaECT | 15.14 | 0.4377 |
| Healthy vs. nsCaECT | 8.489 | >0.9999 |
| Healthy vs. nsCaECT bipolar | 17.94 | 0.2304 |
| CTRL vs. µsCaECT | -20.58 | 0.0133 |
| CTRL vs. nsCaECT | -27.24 | 0.0002 |
| CTRL vs. nsCaECT bipolar | -17.78 | 0.0953 |
| µsCaECT vs. nsCaECT | -6.654 | >0.9999 |
| µsCaECT vs. nsCaECT bipolar | 2.8 | >0.9999 |
| nsCaECT vs. nsCaECT bipolar | 9.454 | >0.9999 |
|  |  |  |
| Plasma cells |  |  |
| Dunn's multiple comparisons test | Mean rank diff. | Adjusted P Value |
| Healthy vs. CTRL | -34.78 | <0.0001 |
| Healthy vs. µsCaECT | -15.53 | 0.3859 |
| Healthy vs. nsCaECT | -11.49 | >0.9999 |
| Healthy vs. nsCaECT bipolar | -17.02 | 0.3106 |
| CTRL vs. µsCaECT | 19.25 | 0.0269 |
| CTRL vs. nsCaECT | 23.29 | 0.0028 |
| CTRL vs. nsCaECT bipolar | 17.76 | 0.0962 |
| µsCaECT vs. nsCaECT | 4.038 | >0.9999 |
| µsCaECT vs. nsCaECT bipolar | -1.488 | >0.9999 |
| nsCaECT vs. nsCaECT bipolar | -5.527 | >0.9999 |
|  |  |  |
|  |  |  |
| From Lymph nodes |  |  |
|  |  |  |
| CD3^+^ |  |  |
| Dunn's multiple comparisons test | Mean rank diff. | Adjusted P Value |
| Healthy vs. CTRL | 26.28 | 0.0056 |
| Healthy vs. μsCaEP | 29.76 | 0.0007 |
| Healthy vs. nsCaEP | 21.49 | 0.0421 |
| Healthy vs. nsCaEP bipolar | 25.97 | 0.01 |
| CTRL vs. μsCaEP | 3.484 | >0.9999 |
| CTRL vs. nsCaEP | -4.785 | >0.9999 |
| CTRL vs. nsCaEP bipolar | -0.3083 | >0.9999 |
| μsCaEP vs. nsCaEP | -8.269 | >0.9999 |
| μsCaEP vs. nsCaEP bipolar | -3.792 | >0.9999 |
| nsCaEP vs. nsCaEP bipolar | 4.477 | >0.9999 |
|  |  |  |
| CD4^+^ |  |  |
| Dunn's multiple comparisons test | Mean rank diff. | Adjusted P Value |
| Healthy vs. CTRL | 27.04 | 0.003 |
| Healthy vs. μsCaEP | 13.67 | 0.6378 |
| Healthy vs. nsCaEP | 4.286 | >0.9999 |
| Healthy vs. nsCaEP bipolar | -3.27 | >0.9999 |
| CTRL vs. μsCaEP | -13.37 | 0.338 |
| CTRL vs. nsCaEP | -22.75 | 0.003 |
| CTRL vs. nsCaEP bipolar | -30.31 | 0.0001 |
| μsCaEP vs. nsCaEP | -9.385 | >0.9999 |
| μsCaEP vs. nsCaEP bipolar | -16.94 | 0.1301 |
| nsCaEP vs. nsCaEP bipolar | -7.556 | >0.9999 |
|  |  |  |
| Tregs form CD4^+^ |  |  |
| Dunn's multiple comparisons test | Mean rank diff. | Adjusted P Value |
| Healthy vs. CTRL | -25.3 | 0.0072 |
| Healthy vs. μsCaEP | -10.12 | >0.9999 |
| Healthy vs. nsCaEP | -2.698 | >0.9999 |
| Healthy vs. nsCaEP bipolar | 1.683 | >0.9999 |
| CTRL vs. μsCaEP | 15.18 | 0.1591 |
| CTRL vs. nsCaEP | 22.61 | 0.0033 |
| CTRL vs. nsCaEP bipolar | 26.99 | 0.001 |
| μsCaEP vs. nsCaEP | 7.423 | >0.9999 |
| μsCaEP vs. nsCaEP bipolar | 11.8 | 0.8357 |
| nsCaEP vs. nsCaEP bipolar | 4.38 | >0.9999 |
|  |  |  |
| CD8^+^ cells |  |  |
| Dunn's multiple comparisons test | Mean rank diff. | Adjusted P Value |
| Healthy vs. CTRL | -25.18 | 0.0095 |
| Healthy vs. μsCaEP | -9.643 | >0.9999 |
| Healthy vs. nsCaEP | -0.2967 | >0.9999 |
| Healthy vs. nsCaEP bipolar | 3.857 | >0.9999 |
| CTRL vs. μsCaEP | 15.54 | 0.1536 |
| CTRL vs. nsCaEP | 24.89 | 0.001 |
| CTRL vs. nsCaEP bipolar | 29.04 | 0.0002 |
| μsCaEP vs. nsCaEP | 9.346 | >0.9999 |
| μsCaEP vs. nsCaEP bipolar | 13.5 | 0.451 |
| nsCaEP vs. nsCaEP bipolar | 4.154 | >0.9999 |
|  |  |  |
| B cells |  |  |
| Dunn's multiple comparisons test | Mean rank diff. | Adjusted P Value |
| Healthy vs. CTRL | 8.708 | >0.9999 |
| Healthy vs. µsCaECT | -11.18 | >0.9999 |
| Healthy vs. nsCaECT | -15.56 | 0.4494 |
| Healthy vs. nsCaECT bipolar | -9.883 | >0.9999 |
| CTRL vs. µsCaECT | -19.89 | 0.0158 |
| CTRL vs. nsCaECT | -24.27 | 0.0012 |
| CTRL vs. nsCaECT bipolar | -18.59 | 0.0576 |
| µsCaECT vs. nsCaECT | -4.385 | >0.9999 |
| µsCaECT vs. nsCaECT bipolar | 1.296 | >0.9999 |
| nsCaECT vs. nsCaECT bipolar | 5.681 | >0.9999 |
|  |  |  |
| Plasma cells |  |  |
| Dunn's multiple comparisons test | Mean rank diff. | Adjusted P Value |
| Healthy vs. CTRL | -6.167 | >0.9999 |
| Healthy vs. µsCaECT | 11.96 | >0.9999 |
| Healthy vs. nsCaECT | 11.85 | >0.9999 |
| Healthy vs. nsCaECT bipolar | 12.83 | >0.9999 |
| CTRL vs. µsCaECT | 18.13 | 0.0399 |
| CTRL vs. nsCaECT | 18.01 | 0.0423 |
| CTRL vs. nsCaECT bipolar | 19 | 0.0616 |
| µsCaECT vs. nsCaECT | -0.1154 | >0.9999 |
| µsCaECT vs. nsCaECT bipolar | 0.8718 | >0.9999 |
| nsCaECT vs. nsCaECT bipolar | 0.9872 | >0.9999 |
|  |  |  |
| MDSCs |  |  |
| Dunn's multiple comparisons test | Mean rank diff. | Adjusted P Value |
| Healthy vs. CTRL | -32.14 | 0.0002 |
| Healthy vs. µsCaECT | -7.335 | >0.9999 |
| Healthy vs. nsCaECT | -7.066 | >0.9999 |
| Healthy vs. nsCaECT bipolar | -14.99 | 0.5752 |
| CTRL vs. µsCaECT | 24.81 | 0.0011 |
| CTRL vs. nsCaECT | 25.08 | 0.0009 |
| CTRL vs. nsCaECT bipolar | 17.15 | 0.124 |
| µsCaECT vs. nsCaECT | 0.2692 | >0.9999 |
| µsCaECT vs. nsCaECT bipolar | -7.658 | >0.9999 |
| nsCaECT vs. nsCaECT bipolar | -7.927 | >0.9999 |
